# Integrated RNA-Seq and Metabolomics Analyses of Biological Processes and Metabolic Pathways Involved in Seed Development in *Arachis hypogaea* L.

**DOI:** 10.3390/genes16030300

**Published:** 2025-02-28

**Authors:** Long Li, Yutong Wang, Xiaorui Jin, Qinglin Meng, Zhihui Zhao, Lifeng Liu

**Affiliations:** State Key Laboratory of North China Crop Improvement and Regulation, North China Key Laboratory for Crop Germplasm Resources, Ministry of Education, College of Agronomy, Hebei Agricultural University, Baoding 071001, China; 18513581472@163.com (L.L.); wangyutong1220@163.com (Y.W.); 13932834446@163.com (X.J.); 15931832702@163.com (Q.M.); m15081392769@163.com (Z.Z.)

**Keywords:** peanut, quality and yield, flower, aerpeg, transcriptome, metabolome

## Abstract

In peanut cultivation, fertility and seed development are essential for fruit quality and yield, while pod number per plant, seed number per pod, kernel weight, and seed size are indicators of peanut yield. In this study, metabolomic and RNA-seq analyses were conducted on the flowers and aerial pegs (aerpegs) of two peanut cultivars JNH3 (Jinonghei) and SLH (Silihong), respectively. Compared with SLH, JNH3 had 3840 up-regulated flower-specific differentially expressed genes (DEGs) and 5890 up-regulated aerpeg-specific DEGs. Compared with the JNH3 aerpegs, there were 4079 up-regulated variety-specific DEGs and 18 up-regulated differentially accumulated metabolites (DAMs) of JNH3 flowers, while there were 3732 up-regulated variety-specific DEGs and 48 up-regulated DAMs in SLH flowers. Gene Ontology (GO) and Kyoto Encyclopedia of Genes and Genomes (KEGG) enrichment analyses revealed that the DEGs of JNH3 were associated with pollen germination and phenylalanine metabolism in flower and aerpeg tissues, respectively. In contrast, the DEGs of SLH were associated with protein degradation, amino acid metabolism, and DNA repair. However, there were significant differences in the lipids and lipid-like molecules between JNH3 flowers and SLH flowers. This investigation provides candidate genes and an experimental basis for the further improvement of high-quality and high-yield peanut varieties.

## 1. Introduction

The peanut is a common oil crop and cash crop, and its seed is an important source of oil and nutrition that determines both crop yield and quality [1]. Pod number per plant, seed number per pod, kernel weight, and seed size are important indicators of peanut yield [2]. Plant seed formation includes the development of male and female gametophytes, the maturation of germ cells, double fertilization, and embryo development [3]. Additionally, the genes related to signaling transduction or the metabolic pathways involved in reproductive organ formation and development may affect seed number or kernel size [4]. The peanut is a unique crop that blooms above ground and bears pods below ground. Normally, the stem cells of the ovary divide rapidly and extend to form an aerial peg, or aerpeg, after double fertilization. When the aerpeg enters the soil, seed development starts near the ovary stalk [5,6]. Therefore, identifying the genes regulating seed development from flowers to aerpegs, before the fruits develop in the soil, is important for the genetic improvement of peanut varieties.

Long-term natural selection and domestication have resulted in the genetic diversity of crop germplasm resources [7]. Determining the genetic basis of seed development, as a quantitative genetic trait, is dependent on mapping population construction, genetic loci, and linkage and/or association map analysis [8]. For example, through the analysis of natural population variation and QTLs of *Arabidopsis thaliana*, the ER gene encoding LRR receptor kinase and its receptor EPF/EPFL jointly were found to accurately regulate ovule primordium spacing and affect both ovule number and fruit length [9]. Additionally, the genetic analysis of natural variation was conducted for lines with different numbers of seeds, and it was hypothesized that natural variation in seed number is primarily regulated by maternal effects [10,11]. Through the genome-wide association and T-DNA insertion line phenotypic screening of candidate genes, the root dwarfing new hadron (*NERD1*) gene was identified as possessing a positive regulatory effect on ovule number and male and female gametophyte development [12].

The functional validation of *Arabidopsis* mutants showed that multiple genes related to auxin (AUX), cytokinin (CK), brassinolide (BR), and gibberellin (GA) biosynthesis and signaling pathways (signal transduction, biosynthesis, transport, homeostasis, and degradation) were identified to play important roles in determining pollen fertility, ovule number, and seed number [13,14,15,16]. Several genes involved in auxin metabolism and related pathways have been shown to regulate female reproductive tissue, stamen development, and ovule number, such as *YUC*, a gene encoded a flavin-binding monooxygenase; *ARF5*, *ARF3*, and *SEU*, which encode auxin signaling auxin response factors; *PIN1*, *a* transmembrane auxin transporter gene; and a variety of genes that encode downstream transcription factors, such as *CUC1*, *CUC2*, *ANT*, *CRF2*, and *DRN* [17,18,19,20,21]. The cytokinin oxidase/dehydrogenase gene *CKX*, members of the *AP2/ERF* transcription factor gene family, cytokinin response factor (*CRF*) genes, CK signal transduction response regulator *ARR* genes, the CK receptor histidine kinase genes *CUC1* and *CUC2*, and *UGT85A3* and *UGT73C1*, which are involved in CK synthesis and signal transduction, are all also involved in ovule development [22,23]. Genes involved in BR synthesis and the BR signaling pathway, including *CYP85A2* and *DET2*, the BR receptor genes *BRI1* and *BIN2*, which negatively regulate BR signaling, and the *SIN2* gene encoding mitochondrial DAR GTPase that can be regulated by BRs, participate in reproductive development and affect the number of ovules and seeds [24,25,26].

Limitations in the development of mapping populations and markers in peanuts and the lack of a mature stable genetic transformation system have led to slow progress in the identification of genes. In order to shorten the research cycle and reduce labor, in this study, we utilized JNH3 and SLH peanut varieties as research materials. Transcriptomic and metabolomic analyses were used to detect the differentially expressed genes (DEGs) and differentially accumulated metabolites (DAMs) of JNH3 and SLH in flower and aerpeg tissues, respectively. These results advance the current understanding of the molecular regulation of seed formation and development and provide further candidate genes for verification and genetic breeding.

## 2. Materials and Methods

### 2.1. Materials and Plant Samples

In this study, two peanut varieties of *Arachis hypogaea,* JNH3 and SLH, with significant differences in yield traits and growth period were selected for comparative analysis of transcriptomics and metabolomics for mining genes related to peanut seed development. JNH3, as a late flowering and late maturing variety, has more pods per plant, less seeds per pod, higher 1000-grain weight, larger seeds, and greater disease resistance compared with SLH. JNH3 and SLH were planted in the same field (Hebei Agricultural University, Baoding, China), and the flowers and pods were used as experimental materials. The same field management was used for both varieties during the entire growth period to reduce the impact of environmental factors. Two copies of three flower and peg samples were collected, immediately frozen in liquid nitrogen, and stored at −80 °C until subsequent, separate RNA-seq and metabolome analysis. 

### 2.2. RNA Extraction and Library Construction

The samples used for RNA-seq were collected in September 2023. Total RNA was isolated by means of an RNA purification kit (Tiangen Biotech, Beijing, China) from one copy. Then, RNA quality was determined by a 5300 Bioanalyser (Agilent, Santa Clara, CA, USA) and quantified using the ND-2000 (NanoDrop Technologies Wilmington, DE, USA). Only high-quality RNA samples (OD260/280 = 1.8~2.2, OD260/230 ≥ 2.0, RQN ≥ 6.5, 28S:18S ≥ 1.0, >1 μg) were used to construct the sequencing library. All 12 libraries (4 samples with 3 biological replicates) were multiplex sequenced on the Illumina Nova-Seq 6000 platform (Illumina, San Diego, CA, USA). Poly(A) mRNA sequences were removed from the RNA libraries using oligo-dT-attached magnetic beads. Double-stranded cDNA was synthesized using a Super Script Double-Stranded cDNA Synthesis Kit (Invitrogen, Carlsbad, CA, USA) with random hexamer primers (Illumina) using the short fragments as templates, according to a previously described method [27].

Then the synthesized cDNA was subjected to end repair, phosphorylation, and adapter addition according to library construction protocol. Libraries were size selected for cDNA target fragments of 300 bp on 2% Low Range Ultra Agarose followed by PCR amplified using Phusion DNA polymerase (NEB) for 15 PCR cycles. After being quantified by Qubit 4.0, the sequencing library was performed on the Illumina Nova-Seq 6000 platform (PE150). The raw paired-end reads were trimmed and quality controlled by fastp [28] with default parameters. Then clean data from the samples were used to perform de novo assembly with Trinity [29]. To increase the assembly quality, all the assembled sequences were filtered by CD-HIT [3] and TransRate [30] and assessed with BUSCO (Benchmarking Universal Single-Copy Orthologs) [31]. Next, the assembled high-quality transcripts were aligned to the peanut reference genome (https://data.legumeinfo.org/Arachis/hypogaea/genomes/Tifrunner.gnm2.J5K5/, accessed on 5 October 2023) using HISAT (v2.0.0).

### 2.3. DEG Analysis and Functional Enrichment

The expression level of all genes was calculated according to the transcripts per million reads (TPM) method. TRSEM [32] was used to quantify gene abundances. Differential expression analysis was performed using the DESeq2 (v1.18.1) [33]. The differentially expressed genes of at least|log2fold change| ≥ 1 and a *p*-value < 0.05 were considered to be significantly differentially expressed genes (DEGs). In addition, functional enrichment analyses, including GO and KEGG, were performed to identify which DEGs were significantly enriched in GO terms and metabolic pathways at a Bonferroni-corrected *p*-value < 0.05 were compared with the whole transcriptome background. Gene ontology (GO) and the Kyoto Encyclopedia of Genes and Genomes (KEGG) analyses were performed using OmicShare (http://www.omicshare.com/tools) and OmicStudio (https://www.omicstudio.cn/tool), two free online data analysis platforms [34,35].

### 2.4. Separation of Metabolites from Tissue and GC–MS Analysis

Metabolite extractions were performed using the flower and peg tissues of two peanut varieties from another copy (three biological replicates per sample). For each replicate, flower and peg tissues were separately combined with methanol, following a previously described method with minor improvements [36]. Finally, the samples were analyzed using gas chromatography–mass spectrometry (GC–MS). Qualitative and quantitative analyses of metabolites were conducted using the previously reported method [37]. All metabolite variables were normalized and subjected to PCA. Next, variable importance projection (VIP) values were calculated in an OPLS-DA model, the metabolites with |log2 (fold change)| ≥ 1 and *p*-value < 0.05 were determined as differentially accumulated metabolites (DAMs). DAMs were mapped to pathways through metabolic enrichment analysis (KEGG; http://www.genome.jp/kegg/) and performed using OmicShare (http://www.omicshare.com/tools) and OmicStudio (https://www.omicstudio.cn/tool).

### 2.5. qRT-PCR Analysis

RNA was extracted from the flowers and aerpegs using an RNA extraction kit (Tiangen Biotech, Beijing, China). The primers were designed with Primer5.0 software (Table S1). The cDNA sequences were identified from Peanutbase (https://www.peanutbase.org). Real-time PCR was performed using ChamQ Blue Universal SYBR qPCR Master Mix (Vazyme, Nanjing, China) on a Bio-Rad system (Bio-Rad, Hercules, CA, USA). Relative gene expression was calculated using the 2^−ΔΔCt^ method [38].

### 2.6. Integrated Analysis Between Transcriptome Data and Metabolites

Transcriptomic and metabolomic data were uniformly normalized by Log2 transformation. PCA was carried out on transcriptomes and metabolomes to visually show whether there are differences between the sample groups in transcriptomes and metabolomes, respectively. Correlation analysis was performed on the genes and metabolites detected in each different group. The Pearson correlation coefficients of genes and metabolites were calculated using the core program in R language, and the different gene metabolites with Pearson correlation coefficients greater than 0.8 in each different group were displayed. The correlation analysis of different genes and metabolites was carried out, and the results with Pearson correlation coefficients greater than 0.8 were finally selected.

### 2.7. Data Analysis

Data analysis was performed using SPSS software (version 28; IBM, Armonk, NY, USA) and Origin version 8.0 (OriginLab, Northampton, MA, USA). The results are presented as mean ± SD values of three independent biological replicates.

## 3. Results

### 3.1. Agronomic Trait Summary of the Two Peanut Varieties at the Reproductive Stage

**Figure 1:**
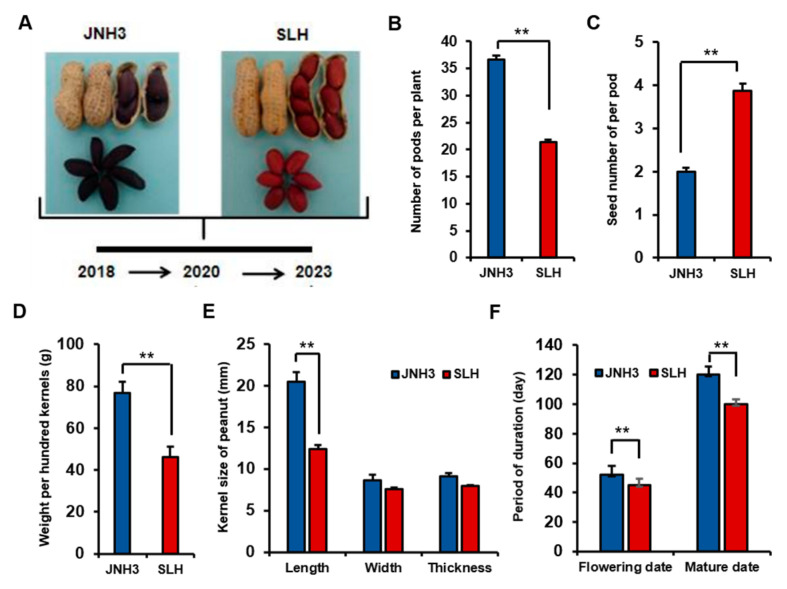
Comparison of important agronomic traits in the breeding stage of the JNH3 and SLH peanut varieties. (**A**) Comparative image of pod and seed characteristics of the two peanut varieties. (**B**) Pod numbers per plant of the two cultivars. (**C**) The number of seeds per pod of the two cultivars. (**D**) One-hundred-kernel weight of the two cultivars. (**E**) Kernel size of the two cultivars. (**F**) Flowering and ripening time of the two cultivars. Note, there were three biological replicates and three experimental replicates for all traits shown. The values of all traits are represented as the mean and standard deviation; **, *p* < 0.01.

Jinonghei 3 (JNH3) is an artificially improved peanut variety with a black seed coat, and most of its fruit produce double kernels. Silihong (SLH) is a local variety grown in northeast China with a deep red seed coat, and about 50% of its pods have three to four seeds per pod (Figure 1A). We compared the main agronomic traits of the two peanut varieties during the breeding stage, which determines the yield of the peanut, and found that in addition to seed coat color differences, there were also significant differences in the number of pods per plant, the number of seeds per pod, 100-kernel weight, kernel size, flowering time, and ripening time (Figure 1A–F). JNH3 cultivar crops planted in 2018, 2020, and 2023 were harvested. Three biological replicates were evaluated for each agronomic trait across fifteen lines. First, the numbers of pods per plant for JNH3 and SLH were 36.70 ± 0.734 and 21.40 ± 0.428, respectively (Figure 1B). The number of pods per plant was significantly higher in JNH3 than SLH (*p* < 0.01). Second, SLH had significantly more single pod seeds than did JNH3 (*p* < 0.01, 2.0 ± 0.089 and 3.87 ± 0.156, respectively; Figure 1C), indicating a negative correlation between the number of pods per plant and number of single pod seeds. Additionally, the 100-kernel weight of the two varieties was also compared, revealing that JNH3 possessed a higher 100-kernel weight at 76.80 ± 5.54 g than that of SLH at 46.20 ± 4.82 g (Figure 1D). Similarly, the seed length of JNH3, at 20.51 ± 1.14 mm, was significantly longer than that of SLH, at 12.42 ± 0.43 mm (*p* < 0.01); however, there were no significant differences in the width or thickness of the seeds between the two varieties (Figure 1E). Therefore, it was hypothesized that the difference in kernel length might underlie the difference in kernel weight between the two varieties. Finally, this work demonstrated that the flowering and ripening times of JNH2, at 50 ± 4.5 d and 120 ± 6 d, respectively, were significantly longer than those of SLH, at 45 ± 3.4 d and 100 ± 5.4 d, respectively (Figure 1F), indicating that JNH3 is a late-flowering and late-maturing peanut variety. To analyze the gene regulatory and metabolic mechanisms of the differences in agronomic traits between JNH3 and SLH in the breeding stage, transcriptome and metabolome analyses were performed on the flowering organs and aerpegs from the two varieties in this study, with the aim of identifying important genes related to the quality and yield of crops.

### 3.2. RNA-Seq Analysis of the Molecular Changes of Flowers and Aerpegs

**Figure 2:**
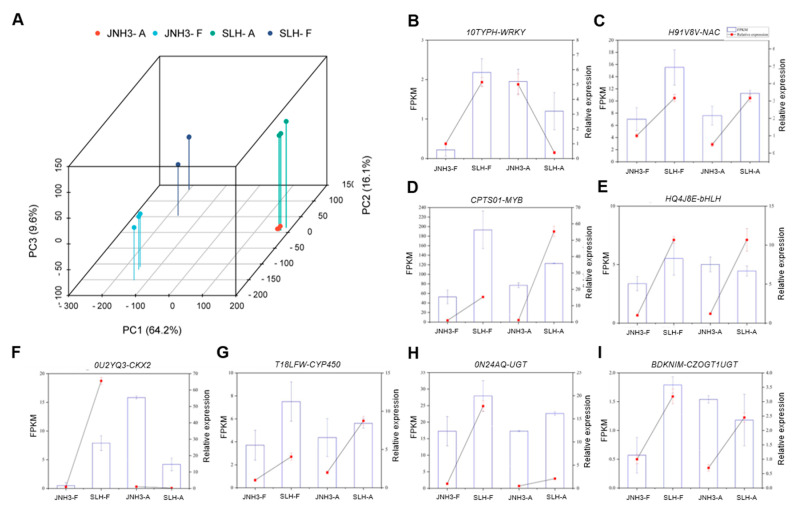
Principal component analysis map and quantitative real-time PCR (qRT-PCR) verification. (**A**) PCA analysis map of JNH3-A, JNH3-F, SLH-A, and SLH-F samples. (**B**–**I**) qRT-PCR verification for eight randomly selected DEGs. The left *y*-axis indicates the relative expression levels as fragments per kilobase of million mapped reads (FPKM) values, and the right *y*-axis indicates the analysis results of qRT-PCR according to 2^−ΔΔCT^ methods. The relative expression of each sample is represented by its mean ± SD value.

RNA-seq analysis was conducted to identify the transcriptomic differences between JNH3 and SLH in their flowers (JNH3-F and SLH-F, respectively) and aerpegs (JNH3-A and SLH-A, respectively). Twelve cDNA libraries were established, including tissues at two developmental stages from the two varieties, with three biological replicates. The three biological replicates of JNH3-F, JNH3-A, SLH-F, and SLH-A produced 55.14 M, 97.95 M, 36.72 M, and 90.56 M clear reads on average, respectively. Quality control analysis indicated that 93.45–95.02% of reads had a Phred quality score of at least Q30, affirming the high quality and suitability of the sequencing data for gene expression analysis. Transcripts were annotated by referencing the KEGG, Swiss-Prot, Pfam, GO, COG, and NR databases. PCA discriminated the twelve samples into four groups, thereby effectively capturing differences among the four groups. PC1, PC2, and PC3 explained 64.2%, 16.1%, and 9.6% of the variation, respectively (Figure 2A). The results indicated the reliability of the replication within each group and highlighted obvious differences between the two tissues and two varieties. To verify the accuracy of the DEGs determined by RNA-seq, quantitative real-time PCR (qRT-PCR) was conducted on eight randomly selected DEGs using identical cDNA templates. These gene expression results exhibited an extremely significant positive correlation with the RNA-seq expression data, indicating the reliability of the transcriptomic results (Figure 2B–I).

### 3.3. Identification of DEGs in Flowers and Aerpegs of Two Peanut Varieties

**Figure 3:**
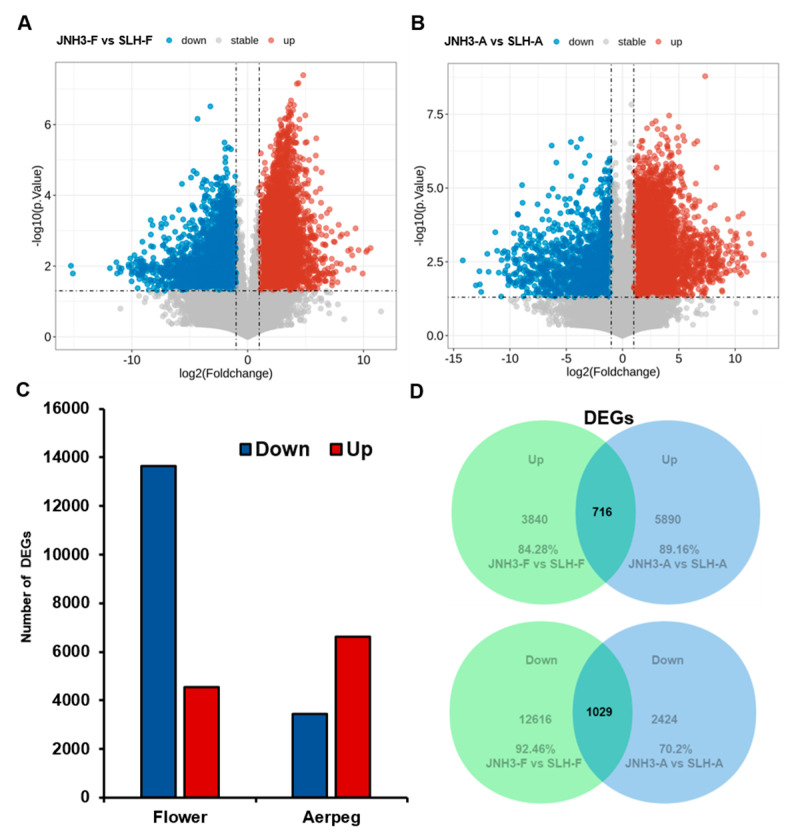
Volcano plot and summary of differentially expressed genes (DEGs) between JNH3 and SLH in the flower and aerpeg tissues. (**A**,**B**) Volcano plot between JNH3 and SLH in flower and aerpeg tissues. (**C**) DEGs between JNH3 and SLH at the flowering and aerpeg stages. (**D**) Venn diagrams of DEGs between JNH3 and SLH at the flowering and aerpeg stages.

The DEGs between the flowers and aerpegs of the two peanut varieties were identified. In the flower samples, JNH3 exhibited 18,201 DEGs (4556 up-regulated and 13,645 down-regulated) compared with SLH (Figure 3A,C), while in the aerpeg samples, JNH3 had 10,059 DEGs (6606 up-regulated and 3453 down-regulated) compared with SLH (Figure 3B,C). Thus, more DEGs were identified in flowers than aerpegs between the two varieties. Venn diagrams of the DEGs between the two peanut varieties across the two different tissues showed that, compared with SLH, JNH3 had 716 overlapping up-regulated DEGs and 1029 overlapping down-regulated DEGs at the flowering and aerpeg stages, respectively. These DEGs may play a prominent role in both developmental stages. According to the Venn diagram, compared with SLH, 3840 flower-specific up-regulated DEGs and 12,616 flower-specific down-regulated DEGs were found in JNH3 in flower tissue (Figure 3D, upper panel). In the aerpegs, JNH3 was found to have 5890 aerpeg-specific up-regulated DEGs and 2424 aerpeg-specific down-regulated DEGs (Figure 3D, lower panel).

### 3.4. GO Annotation Analysis of JNH3 and SLH in Flower and Aerpeg Tissues

**Figure 4:**
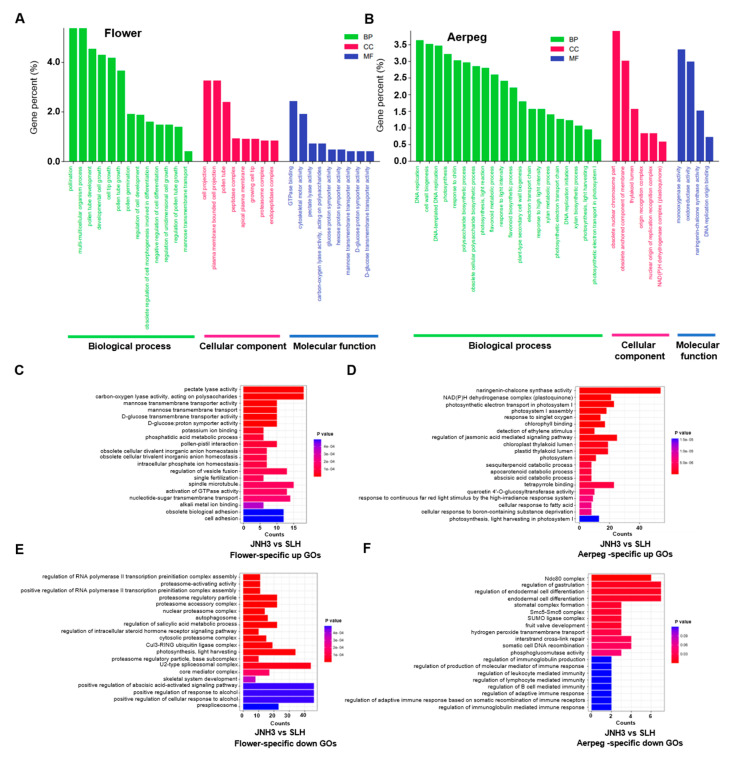
Gene Ontology (GO) functional enrichment analysis of all differentially expressed genes (DEGs) in JNH3 and SLH both in flowers and aerpegs. (**A**) GO enrichment analysis of all DEGs in JNH3 and SLH in flowers. (**B**) GO enrichment analysis of all DEGs in JNH3 and SLH in aerpegs. The *y*-axis represents the gene percentage, and the GO terms are shown along the *x*-axis. The green line, red line, and blue line represent biological processes, cellular components, and molecular functions, respectively. (**C**) The top 20 up-regulated flower-specific enriched GO terms in JNH3 and SLH. (**D**) The top 20 up-regulated aerpeg-specific enriched GO terms in JNH3 and SLH. (**E**) The top 20 down-regulated flower-specific enriched GO terms in JNH3 and SLH. (**F**) The top 20 down-regulated aerpeg-specific enriched GO terms in JNH3 and SLH. The *y*-axis represents the enriched GO terms, and the numbers of GO terms are shown along the *x*-axis.

GO and KEGG functional enrichment analyses were conducted on the DEGs identified in the flower and aerpeg tissues between the two peanut varieties. In short, 4556 up-regulated DEGs and 13,645 down-regulated DEGs in JNH3 flower tissues were categorized into 5792 GO entries in the GO database and compared with SLH. Pollination (GO:0009856), multi-multicellular organism process (GO:0044706), and pollen tube development (GO:0048868) were the most enriched biological process (BP) GO terms (Figure 4A). Furthermore, cell projection (GO:0042995), plasma membrane-bounded cell projection (GO:0120025), and pollen tube (GO:0090406) were the most enriched cellular component (CC) GO terms (Figure 4A). GTPase binding (GO:0051020), cytoskeletal motor activity (GO:0003774), and pectate lyase activity (GO:0030570) were the most enriched molecular function (MF) GO terms (Figure 4A).

Meanwhile, 6606 up-regulated DEGs and 3453 down-regulated DEGs in JNH3 aerpegs corresponded to 5344 GO entries in the GO database compared with SLH. DNA replication (GO:0006261), cell wall biogenesis (GO:0042546), and DNA-templated DNA replication (GO:0006261) were the most significantly enriched BP GO terms. Obsolete nuclear chromosome part (GO:0044454) and obsolete anchored component of membrane (GO:0031225) were the most significantly enriched CC GO terms. Monooxygenase activity (GO:0004497) and oxidoreductase activity (GO:0016646) were the most enriched MF GO terms (Figure 4B). The GO terms identified as enriched among the DEGs of the two peanut varieties at different developmental stages were compared. As visualized in a Venn diagram, 3794 up-regulated GOs and 4222 down-regulated GOs were found in JNH3 compared with SLH at the flowering and aerpeg stages. Compared with SLH, there were 699 flower-specific up-regulated enriched GO terms in JNH3 at the flowering stage, which were mainly comprised of pectate lyase activity, carbon-oxygen lyase activity, acting on polysaccharides, mannose transmembrane transporter activity, d-glucose: proton symporter activity, pollen–pistil interaction, and single fertilization (Figure 4C). At the same time, there were 1446 flower-specific enriched GO terms down-regulated in JNH3 at the flowering stage, mainly comprising proteasome-activating activity, proteasome regulatory particle, and proteasome accessory complex (Figure 4D). Compared with SLH, JNH3 had 803 aerpeg-specific up-regulated enriched GO terms in aerpegs, mainly comprising naringenin-chalcone synthase activity, sesquiterpenoid catabolic process, apocarotenoid catabolic process, and tetrapyrrole binding (Figure 4E). There were also 82 GO terms enriched for aerpeg-specific down-regulated DEGs, mainly concentrated in fruit valve development, Ndc80 complex, regulation of gastrulation, regulation of endodermal cell differentiation, and endodermal cell differentiation (Figure 4F).

### 3.5. Heatmaps of Organ-Specific DEGs and KEGG Pathway Enrichment

**Figure 5:**
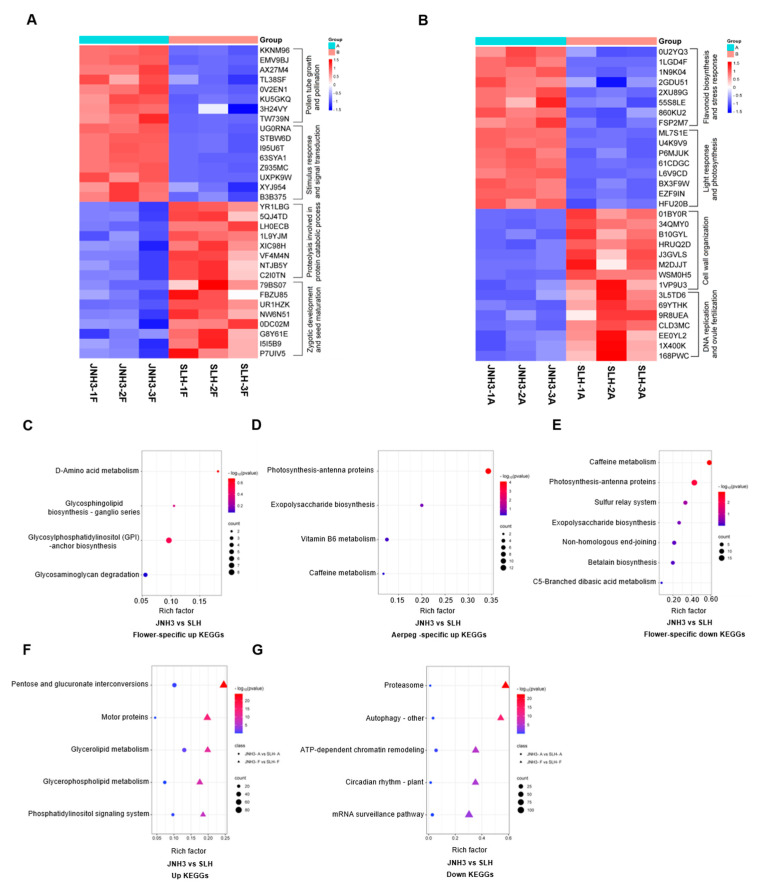
Heatmaps of differentially expressed genes (DEGs) and Kyoto Encyclopedia of Genes and Genomes (KEGG) pathway enrichment. (**A**) Heatmap of DEGs based on tissue-specific Gene Ontology (GO) terms in flowers. (**B**) Heatmap of DEGs based on tissue-specific GO terms in aerpegs. (**C**) Up-regulated flower-specific KEGG pathway enrichment in JNH3 and SLH. (**D**) Up-regulated aerpeg-specific KEGG pathway enrichment in JNH3 and SLH. (**E**) Down-regulated flower-specific KEGG pathway enrichment in JNH3 and SLH. (**F**) Up-regulated overlapped KEGG pathway enrichment in JNH3 and SLH both in flower and aerpeg tissues. (**G**) Down-regulated overlapped KEGG pathway enrichment in JNH3 and SLH both in flower and aerpeg tissues.

According to the tissue-specific DEG GO terms identified above, we found corresponding DEGs. Cluster analysis of these DEGs was performed, and heat maps were used to visualize the expression patterns of these DEGs in plant tissues. In flowers, compared to SLH, genes related to pollen germination and pollen tube elongation, external response, and signal transduction were significantly up-regulated in JNH3. Genes related to zygotic development and seed maturation, and protein degradation regulatory genes that participate in protein catalysis were obviously down-regulated in JNH3 (Figure 5A). In aerpegs, compared to SLH, genes related to flavonoid biosynthesis and stress response regulatory response, light response, and photosynthesis regulatory response were significantly up-regulated in JNH3 (Figure 5B). However, regulatory genes related to cell wall synthesis, DNA repair, and ovule fertility were significantly down-regulated in JNH3.

To better understand the biological function of these obviously enriched DEGs, KEGG pathway enrichment analysis was used, showing that JNH3 had four significantly up-regulated flower-specific enriched KEGG pathways compared to SLH: glycosylphosphatidylinositol (GPI)-anchor biosynthesis, glycosphingolipid biosynthesis—ganglio series, d-amino acid metabolism, and glycosaminoglycan degradation (Figure 5C). At the same time, JNH3 also had seven significantly down-regulated flower-specific enriched KEGG pathways: caffeine metabolism, photosynthesis-antenna proteins, sulfur relay system, exopolysaccharide biosynthesis, non-homologous end-joining, betalain biosynthesis, and C5-dranched dibasic acid metabolism (Figure 5D). In the aerpegs, JNH3 had four up-regulated aerpeg-specific enriched KEGG pathways: photosynthesis-antenna proteins, exopolysaccharide biosynthesis, vitamin B6 metabolism, and caffeine metabolism (Figure 5E). However, there were KEGG pathways enriched for down-regulated aerpeg-specific DEGs in JNH3. Finally, the overlapping KEGGs of the two varieties at different periods were analyzed, and it was found that compared with SLH, JNH3 had 118 overlapping up-regulated and 119 down-regulated enriched KEGG pathways both in flower and aerpeg tissues. The top five up-regulated enriched KEGG pathways were pentose and glucuronate interconversions, motor proteins, glycerolipid metabolism, glycerophospholipid metabolism, and phosphatidylinositol signaling system (Figure 5F). The top five down-regulated enriched KEGG pathways included proteasome, autophagy-other, ATP-dependent chromatin remodeling, circadian rhythm–plant, and mRNA surveillance pathway (Figure 5G).

### 3.6. DEGs, GO Terms, and KEGG Pathway Enrichment Characteristics of the Same Peanut Variety in Different Periods

**Figure 6:**
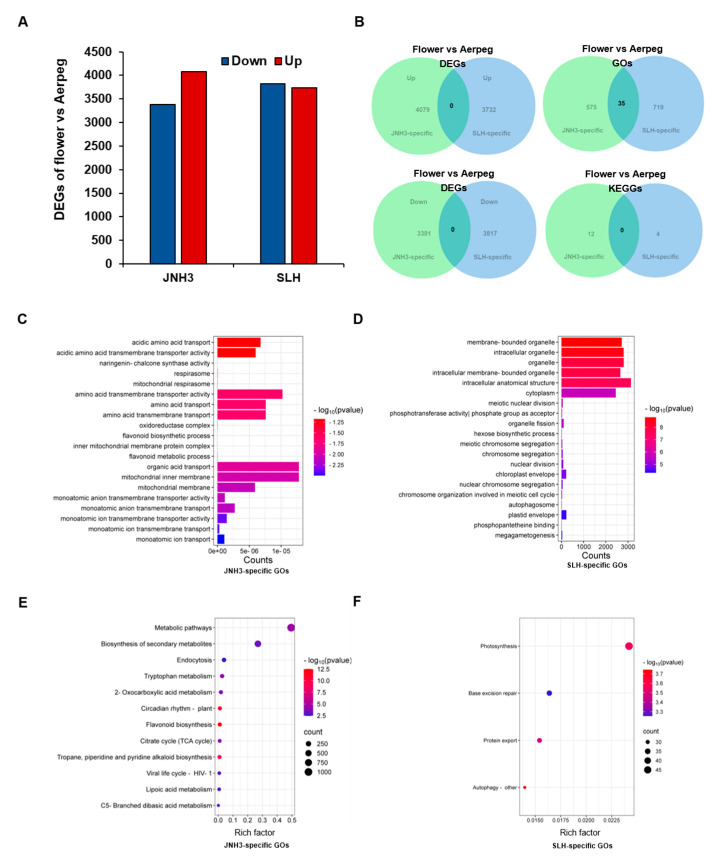
Heatmaps of differentially expressed genes (DEGs) and Kyoto Encyclopedia of Genes and Genomes (KEGG) enrichment. (**A**) Heatmap of DEGs based on tissue-specific enriched Gene Ontology (GO) terms in flower tissue. (**B**) Heatmap of DEGs based on tissue-specific enriched GO terms in aerpeg tissue. (**C**) Up-regulated flower-specific enriched KEGG pathways in JNH3 and SLH. (**D**) Up-regulated aerpeg-specific enriched KEGG pathways in JNH3 and SLH. (**E**) Down-regulated flower-specific enriched KEGG pathways in JNH3 and SLH. (**F**) Up-regulated overlapping enriched KEGG pathways in JNH3 and SLH among DEGs between flower and aerpeg tissues.

According to the analysis of DEGs in the two developmental stages of the same peanut variety, 7460 DEGs were found in JNH3 between the flower and the aerpeg tissues, including 3381 down-regulated DEGs and 4079 up-regulated DEGs (Figure 6A). For SLH, there were 7549 DEGs between flower and aerpeg tissues, including 3917 down-regulated DEGs and 3732 up-regulated DEGs. The DEGs of the two different varieties in two tissues were visualized as Venn diagrams, which showed that there was no overlap between the DEGs of JNH3 and the DEGs of SLH between the flower and aerpeg samples. Thus, the DEGs occurring in the two varieties between the two different tissues were variety-specific DEGs (Figure 6B). At the same time, we determined the enriched GO terms among the DEGs and found 575 variety-specific enriched GO terms for JNH3 between flower and aerpeg tissues (Figure 6B), mainly including acidic amino acid transport, acid amino acid transmembrane transporter activity, and amino acid transmembrane transporter activity, among others (Figure 6C). For SLH, there were 719 variety-specific enriched GO terms between flowers and aerpegs (Figure 6B), mainly including membrane-bounded organelle, intracellular organelle, organelle, and intracellular membrane-bounded organelle, among other terms (Figure 6D). In addition, there were 35 non-variety-specific overlapping enriched GO terms between the two varieties (Figure 6B). Based on GO functional annotation and KEGG metabolic pathway analysis, we also found that 12 variety-specific enriched KEGG pathways existed between the flowering and fruit needling stage for JNH3, including metabolic pathways, biosynthesis of secondary metabolism, tryptophan metabolism, and flavonoid biosynthesis, among other pathways (Figure 6E). There were four variety-specific enriched KEGG pathways in SLH between flower and aerpeg tissues, which were mainly concentrated in photosynthesis, base excision repair, protein export, and autophagy-other pathways (Figure 6F). Moreover, there was no overlap in the enriched KEGG pathways between JNH3 and SLH in the DEGs of flower and aerpeg tissues.

### 3.7. Differential Metabolite Changes and KEGG Pathway Enrichment Within the Same Variety Between Flower and Aerpeg Tissues

**Figure 7:**
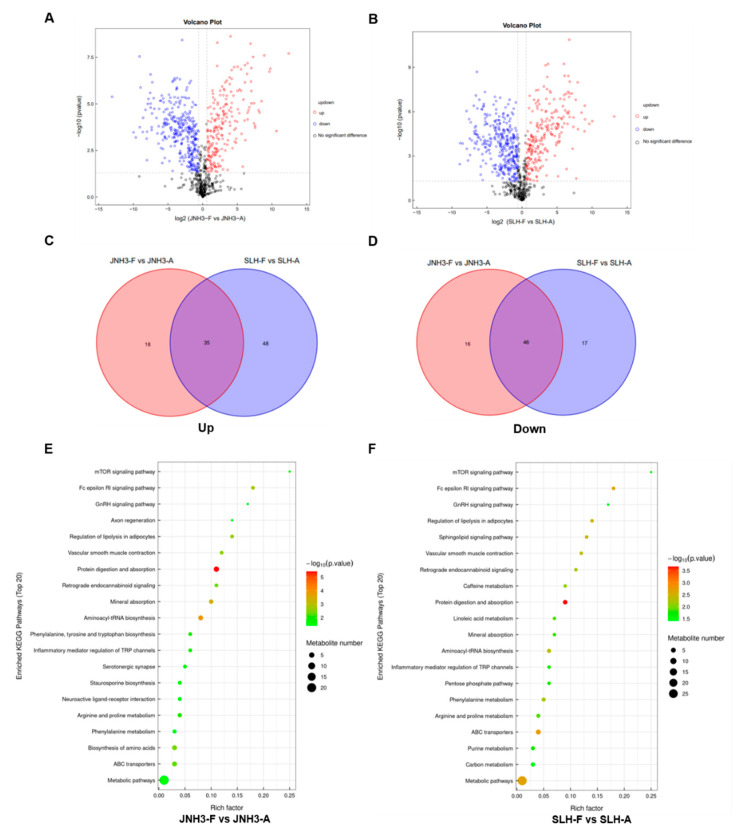
Differentially accumulated metabolites (DAMs) detected in JNH3 and SLH in flower and aerpeg tissues. (**A**) A volcano plot of DAMs in JNH3 flower and aerpeg tissues. (**B**) The volcano plot of DAMs in SLH flower and aerpeg tissues. Red represents up-regulated proteins, blue represents down-regulated proteins, and black represents metabolites that were not significantly changed. (**C**) Venn diagram of the 53 up-regulated DAMs in JNH3 flower and aerpeg tissue and the up-regulated 83 DAMs in SLH flower and aerpeg tissue. (**D**) Venn diagram of the 62 down-regulated DAMs in JNH3 flower and aerpeg tissue and the up-regulated 63 down-regulated DAMs in SLH flower and aerpeg tissue. (**E**,**F**) Kyoto Encyclopedia of Genes and Genomes (KEGG) enrichment analysis of DAMs in JNH3 and SLH flower and aerpeg tissues, respectively. KEGG pathways are shown along the *y*-axis, while the enrichment factor is shown on the *x*-axis.

Comparative metabonomic analyses of flower and aerpeg tissues from JNH3 and SLH plants were performed. In total, 115 differentially accumulated metabolites (DAMs) were identified in JNH3 between flower and aerpeg tissues; the content of 62 metabolites decreased, and the content of 53 metabolites increased (Figure 7A,C). Additionally, 146 DAMs were identified in SLH between flower and aerpeg tissues; the content of 63 metabolites decreased, and the content of 83 metabolites increased (Figure 7B,D). KEGG pathway enrichment analysis identified the top 20 significantly enriched metabolic pathways among DAMs in JNH3 and SLH flower and aerpeg tissues (Figure 7E,F). The top three enriched KEGG pathways among JNH3 DAMs were protein digestion and absorption, aminoacyl-tRNA biosynthesis, and mineral absorption. Phenylalanine, tyrosine, and tryptophan biosynthesis was also enriched among DAMs, and this pathway was closely linked with phenylpropanoid metabolism (Figure 7E). The top three enriched KEGG pathways among SLH DAMs were protein digestion and absorption, metabolic pathways, ABC transporters, and mineral absorption (Figure 7F); furthermore, pathways related to carbon metabolism and the pentose phosphate pathway were also enriched for DAMs, and these pathways included starch and sucrose metabolism and arginine and proline metabolism.

### 3.8. Integrated Analysis of Metabolomic and RNA-Seq Data in JNH3 and SLH in Flower and Aerpeg Tissues

**Figure 8:**
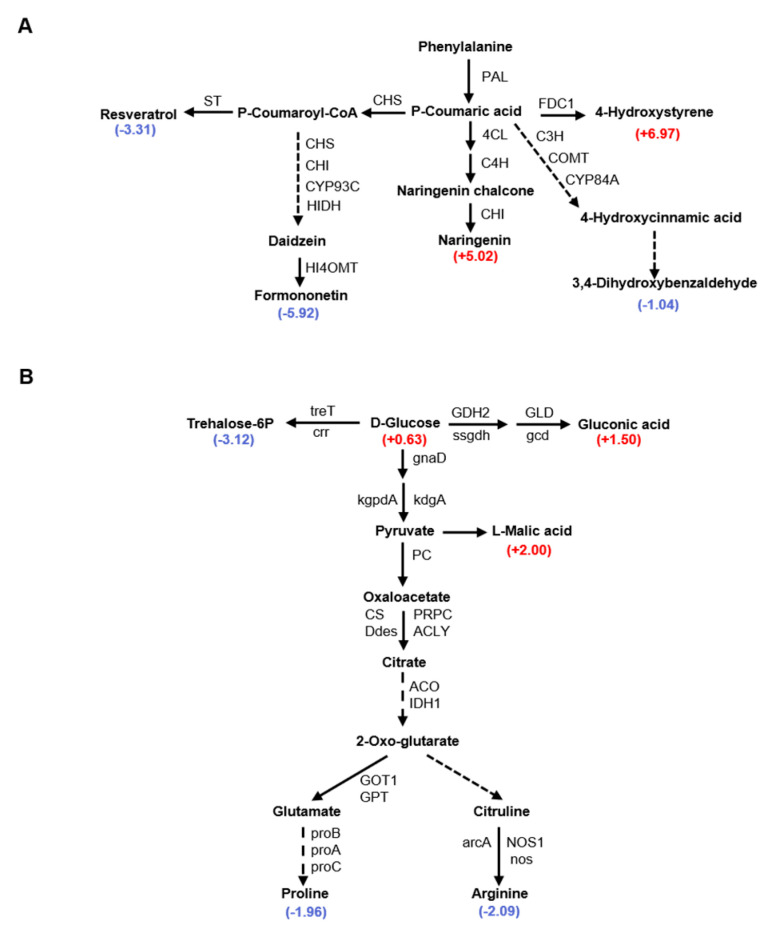
The metabolomics of JNH3 and SLH in flower and aerpeg tissues. (**A**) Phenylpropanoid biosynthesis pathways. (**B**) Starch and sucrose metabolism and arginine and proline metabolism pathways. Each node corresponds to a metabolite, and the number next to the metabolite indicates the log_2_(fold change) value of the metabolite. Red represents up-regulation, and blue represents down-regulation. Pathway numbers indicate the name of the enzyme catalyzing the corresponding reaction.

The merged analysis of the RNA-seq and metabolomic data revealed that phenylpropanoid biosynthesis, flavonoid biosynthesis, isoflavonoid biosynthesis, stilbenoid, diarylheptanoid and gingerol biosynthesis, and isoquinoline alkaloid biosynthesis were significantly enriched among the DEGs and DAMs of JNH3 between flower and aerpeg tissues. Among them, a total of 59 genes involved in flavonoid biosynthesis were enriched, and 25 genes involved in isoflavonoid biosynthesis were DEGs (Figure 8A). In JNH3, the accumulation of resveratrol and pedoflavin in flowers was less than that of aerpegs, while the accumulation of naringin in flowers was higher than that in aerpegs. It is speculated that the common medicinal compounds in these peanuts begin to form during the aerpeg formation period and are transported to the fruit for storage. Similarly, glycolysis/gluconeogenesis, the pentose phosphate pathway, starch and sucrose metabolism, and arginine and proline metabolism were significantly enriched among DEGs in SLH between flower and aerpeg tissues. In these four KEGG metabolic pathways, 72, 18, 56, and 30 significant DEGs were enriched, respectively (Figure 8B). In SLH, the content of fructose-6 phosphate and two amino acids in flowers were lower than those in aerpeg tissues. It is speculated that large amounts of sugars and amino acids accumulate in aerpegs, in preparation for the next steps of seed formation, development, and maturation.

## 4. Discussion

The yield factors and nutritional factors of peanut varieties are very important for modern breeding. In this study, two peanut varieties with obvious differences in seed coat color, seed number per pod, 100-seed weight, seed size, and flowering and maturity stages were subjected to transcriptome sequencing and metabolome analysis and comparison, with the aim of identifying important regulatory genes associated with yield traits and kernel nutrition differences between the two varieties. At the same time, the revealed molecular regulatory mechanisms provide an empirical basis and gene resources for breeding high-yield and high-quality peanut varieties [39].

After the identification of DEGs in the two different varieties within each of the two tissues and GO annotation, some organ-specific or development-specific genes were identified, as shown in Figure 5A,B. The expression patterns of these tissue-specific genes differed between the varieties [40]. Additionally, identifying and exploring genes related to pod size further deepens our understanding of the molecular mechanisms of seed development. During flowering to pod development, pollen activity and ovule number are the key factors affecting double fertilization and seed setting rate [41]. Figure 5A shows that, compared with SLH, JNH3 had many up-regulated genes related to pollen tube growth during the flowering stage, resulting in a significant increase in the number of fruit pods per plant (Figure 1B). Compared with JNH3, a large number of genes related to fertilized zygote development and seed maturation were up-regulated in SLH during flowering (Figure 5A), and genes related to DNA repair and ovule fertility were up-regulated in SLH during the fruiting stage (Figure 5B), which may be related to the early flowering and maturity of SLH [42]. The specific regulatory mechanism of the above characteristics needs more detailed functional verification of the candidate genes to assist elucidation.

The enrichment of DEGs in GO terms and KEGG metabolic pathways contributes to a detailed interpretation of the function of these DEGs. Compared with SLH, there were 699 up-regulated flowering-specific GO terms in JNH3 at the flowering stage. There are many biological processes involved in polysaccharide metabolism, pollen tube formation, and the double fertilization of male and female gametes (Figure 4C). In addition, compared with SLH, there were 803 up-regulated DEGs in enriched GO terms in JNH3, which were mainly concentrated in natural pigment metabolism, photosynthesis, and plant hormone metabolism (Figure 4D). This provides some explanations for the phenomena of the darker seed coat color, larger kernels, greater seed weight, and early fruit maturation of JNH3 than SLH.

Based on the analysis of DEGs, compared with SLH, JNH3 had four KEGG pathways enriched for up-regulated flower-specific DEGs (Figure 5D), which were involved in membrane protein transport, cell adhesion, cell wall synthesis, and surface protection. In yeast, GPI anchoring proteins are components of the cell wall and are necessary for cell integrity [43]. The glycosphingolipid biosynthesis ganglio series metabolic pathway is involved in the composition of important structures of biofilms and plays an important role in signal transduction [44]. Glycosaminoglycans are highly hydrophilic and are affinity agents for polyvalence anions. In aerpegs, compared with SLH, JNH3 had four KEGG pathways that were enriched for up-regulated DEGs (Figure 5D). Small grain mutants in maize, for example, reveal the importance of vitamin B6 biosynthesis for embryo formation and endosperm development in maize [45]. Caffeine metabolism produces alkaloids that resist insects and improve immunity [46].

GO and KEGG enrichment analyses of the same peanut variety at two different developmental stages were analyzed, revealing that JNH3 has 575 variety-specific DEGs enriched within GO terms between flower and aerpeg tissues (Figure 6B). These GO terms may be related to the synthesis and transport of amino acids (Figure 6C); thus, for JNH3, the most obvious difference between the two tissues was associated with amino acids. Based on GO functional annotation and KEGG metabolic pathway analysis, 12 variety-specific enriched KEGG pathways were identified between the flowering and aerpeg stages for JNH3. These KEGG pathways may be involved in various secondary metabolites (Figure 6E), Therefore, in JNH3, the biggest difference identified among enriched KEGG pathways was within the biosynthesis of auxin and secondary metabolites. Thus, the amino acid synthesis and transport functions in the GO entries may be associated with the DEG enrichment of these KEGG pathways [47]. For SLH, there were 719 variety-specific enriched GO terms among DEGs between the flower and the aerpeg tissues (Figure 6B), mainly including virous organelle (Figure 6D). These GO terms may be related to the function of organelles, such as the Golgi apparatus, endoplasmic reticulum, chloroplast, vacuole, and mitochondria. Organelles are generally involved in protein, carbohydrate, and fat synthesis, and secondary metabolites synthesis [48,49,50,51]. Therefore, in SLH, the main difference between the two tissues was likely associated with the function of the various organelles. There were four variety-specific enriched KEGG pathways among DEGs in SLH between flower and aerpeg tissues (Figure 6F), which were mainly involved in photosynthesis, base repair, protein transport, and autophagy. Therefore, in SLH, the biggest difference between the two tissues was associated with photosynthesis and cell clearance autophagy [52,53]. These KEGG pathways are consistent with the biological functions of the identified GO terms.

Transcriptome sequencing and metabolomics are of great importance for the study of peanut development at the flowering stage and pod needling stage [54,55]. The phenylalanine metabolism pathway is an important pathway that affects the content of secondary metabolites and the nutritional quality of seeds [56,57]. A number of studies have shown that resveratrol, naringin, and oncetin are compounds in peanut kernels that are also involved in the resistance to stress, and they are related to the color and cytochrome content of plant tissues [58,59,60]. In this study, transcriptome and metabolome analyses of JNH3 at the flowering and peg initiation stages showed that KEGG pathways highly enriched with DEGs were related to the phenylalanine metabolic pathway and flavonoid anabolic pathway, and the synthesis of secondary metabolites of these metabolic pathways involved multiple genes. Based on transcriptome data, 84 candidate genes related to phenylpropionic acid metabolism were enriched. Studies have shown that starch accumulation and amino acid metabolism generally affect plant seed size and weight. In addition to being a nutrient, starch is also an important factor in peanut seed size and grain weight. In this study, the transcriptome and metabolome at flowering and fruit initiation stages exhibited a high enrichment of KEGG pathways in SLH that were closely related to starch and sucrose metabolism, carbon fixation, and amino acid metabolism. Based on the transcriptome data, 176 genes were determined to be enriched in these metabolic pathways.

## 5. Conclusions

The present study analyzed the molecular mechanisms responsible for the yield and nutrition of two different peanut varieties through transcriptome and metabolome analyses and identified the stage-specific genes of JNH3 and SLH as well as the variety-specific genes that were differentially expressed in the same developmental period. This work provides empirical support and an experimental basis for the further functional verification of candidate genes and marker-assisted breeding of peanut varieties.

## Data Availability

Raw Illumina sequencing reads have been deposited in the Genome Sequence Archive (Genomics, Proteomics & Bioinformatics 2021) in the National Genomics Data Center (Nucleic Acids Res 2024), the China National Center for Bioinformation/Beijing Institute of Genomics, and the Chinese Academy of Sciences (GSA: CRA021078) which are publicly accessible at https://bigd.big.ac.cn/gsa/browse/CRA021078 (accessed on 1 December 2024). All relevant data can be found within the paper and its Supporting Materials.

## Supplementary Materials

The following supporting information can be downloaded at: https://www.mdpi.com/article/10.3390/genes16030300/s1. Table S1: Primer pairs of eight genes for qRT-PCR.
